# Deworming and the immune status of HIV positive pre-antiretroviral therapy individuals in Arba Minch, Chencha and Gidole hospitals, Southern Ethiopia

**DOI:** 10.1186/s13104-015-1461-9

**Published:** 2015-09-28

**Authors:** Ashenafi Abossie, Beyene Petros

**Affiliations:** Medical Laboratory Technology Team, Arba Minch College of Health Sciences, P. O. Box 155, Arba Minch, Ethiopia; Department of Microbial, Cellular and Molecular Biology, College of Natural Sciences, Addis Ababa University, P.O. Box. 1176, Addis Ababa, Ethiopia

**Keywords:** Helminth/HIV co-infection, CD4+ T-cells, Deworming, Pre-ART

## Abstract

**Background:**

Helminths/HIV co-infections are very common in developing countries, especially in Africa. The effect of overlapping distribution of HIV and helminths becomes important because concomitant infection may exacerbate disease outcome of HIV infection. The study aimed at determining the effect of deworming on the immune status of helminth/HIV coinfected Pre-ART HIV patients attending three health institutions in Southern Ethiopia.

**Methods:**

97 HIV-positive Pre-ART individuals were observed into 2 groups on the basis of helminth co-infection and no infection. Out of these, 66 study participants were helminths/HIV co-infected and the remaining 31 study participants were helminths (−)/HIV (+) control. Helminth/HIV co-infected participants CD4+ T-cell count was done at baseline, after 15 weeks and 6 months after antihelminthics treatment. Data were analyzed using SPSS version 16.

**Results:**

*Ascaris lumbricoides* was the highest prevalent soil transmitted helminths in Pre-ART individuals in this study. CD4+ T-cell count in the *Ascaris lumricoides*/HIV co-infected was significantly higher (P = 0.05) and (P < 0.05) after 15 weeks and 6 months post-antihelminthics treatment respectively. Also, after antihelminthic therapy, the CD4+ T-cell count significantly increased (P < 0.005) in all treated helminth infections.

**Conclusions:**

The study showed that treatment of ascariasis had a significant effect on CD4+ T-cell count increase in the treated Pre-ART *Ascaris lumbricoides*/HIV co-infected individuals; whereas the same positive effect was not evident for other intestinal helminth parasites detected in the study. In conclusion, this finding on *Ascaris lumbricoides*-specific nature of immune interaction in helminth/HIV co-infection may partly explain the inconsistent reports on the role of intestinal helminths on progression of HIV infection to AIDS. Therefore, a well-designed longitudinal study on helminth species-specific HIV/helminth co-infection will be needed to fully establish the possible benefits of deworming in intestinal helminth/HIV co-infection.

## Background

It is estimated that approximately one-third (almost over 2 billion people) in developing regions of sub- Saharan Africa, Asia, and the Americas are infected with one or more helminth parasite. Among these, the most commons are soil-transmitted helminths such as *Ascaris lumbricoides*, the hookworms (*Ancylostoma duodenale* and *Necator americanus*) and *Trichuris trichiura* [1].

Intestinal helminths infections are most common in areas with over-population and inadequate sanitation in tropical and subtropical countries, where the climate supports the survival of the parasite eggs or larvae in the warm and moist soil [2]. The infections coincide geographically with devastating microbial infectious diseases, including HIV, tuberculosis and malaria. Individuals infected with intestinal helminthic infection may fail to develop protective immune responses when exposed to unrelated pathogens, such as *Plasmodium*, *Mycobacterium* and HIV [3–5]. Although such infections are ubiquitous in Sub-Saharan countries, their effect on the epidemiology of HIV infection, including the risk of HIV transmission and disease progression and management remains uncertain. The areas have high levels of parasitic infections and geographically HIV infection overlaps with areas in which intestinal helminth infections are also common [6].

On the other hand, intestinal helminth/HIV co-infected individuals induce Th2 type immune response through turning off Th1 response and immune system activation. This shifting of Th2 type and chronic immune activation may increase the risk of acquiring HIV infection and potentiate the virulence of both infections in the co-infected individuals [7]. Furthermore, as a primary target of HIV infection, the decline of CD4+ cells used as a biomarker of progression to AIDS in HIV infected individuals. It has also been suggested that deworming would reduce CD4+ T-cell activation and this should contribute to reduction in HIV infection multiplication. Investigators have reported inconsistent findings about the effect of deworming in the delay of HIV infection progression into AIDS through interruption of immune activation in different parasite species [8–11].

This study attempted to determine the effect of deworming on the count of CD4+ T immune cells in helminth/HIV co-infected Pre-ART HIV positive individuals. The effect of deworming in HIV/helminth co-infected patients in protecting against fast deterioration of CD4+ T-cell count so as to require initiation of anti-retroviral therapy (ART) was also assessed.

## Methodology

### The study areas

The study was conducted from December 2008–September 2009 in Arba Minch, Chencha and Gidole hospitals where there are services of Provider-Initiated HIV Counseling and Testing (PIHCT), Voluntary Counseling and Testing (VCT), ART clinic and laboratory.

Arba Minch town is found in Gamo Gofa zone. It is the largest settlement of zone. It is also located 505 km away from Addis Ababa in southern part of Ethiopia, and 275 km from Hawassa, the regional capital. Based on figures published by the Central Statistical Agency in 2007 [12], the town has a total population of 74,843 and of which 39,192 are males and 35,651 are females. Arba Minch hospital is serving a population of more than 1.5 million. It provides different health service such as outpatients, inpatients, pharmacy, and laboratory with full service, especially for ART purpose. The hospital has been providing HIV VCT service since the early 1990s.

Chencha is a one of a district town of Gamo Gofa zone and located near to Arba Minch, 40 km Northwest of the town. It lies 2500 m above sea level and it is very cold area. Based on figures published by the Central Statistical Agency in 2007 [12], this district has an estimated total population of 111,680 and 13,301 or 11.9 % of its population are urban dwellers, which is greater than the zone average of 9.96 %. The town has a district hospital which provides service such as outpatient, in patient, pharmacy, VCT, PIHCT, ART clinic and laboratory services.

Gidole town is the administrative center of Dirashe special district. It is found 565 km far from Addis Ababa in southern part of Ethiopia. Based on figures from the Central Statistical Agency in 2007 [12], this special district has an estimated total population of 142,678 and 13,178 or 9.23 % of its population are urban dwellers. The town has a district hospital which provides all services for the district community and the surroundings.

### Study participants

All individuals who had helminth/HIV co-infection and whose CD4+ count >200 cells/μl, and who have not been on Highly Active Antiretroviral Therapy (HAART) were included in the study. Furthermore, HIV positive and helminth uninfected Pre- ART individuals whose CD4+ count >200 cells/μl were also included in the study. Since helminth/HIV co-infection individuals whose CD4+ count <200 cells/μ were recommended for ART treatment, they were excluded from this study to observe the effect of deworming on ART treatment naïve individuals. In addition, patients with other health conditions, pregnant women and children less than 8 years-old were excluded due to their status of immunity.

### Sample size determination

**Fig. 1:**
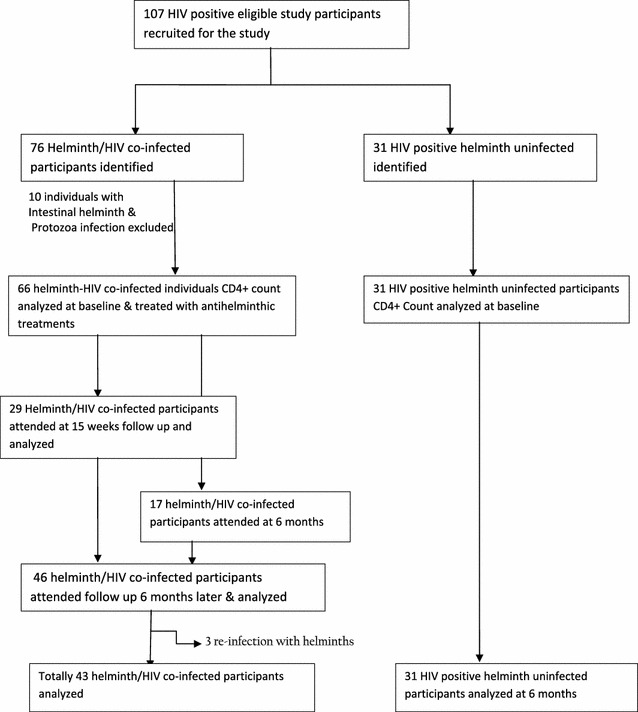
Flow chart of study participants

107 HIV-positive Pre-ART study participants were eligible for this study. 76 of study participants were helminth/HIV co-infected and 31 were HIV-positive helminth uninfected. Excluding mixed infections of intestinal helminth and protozoa the remaining 97 HIV-positive Pre-ART study participants were recruited into 2 study groups on the basis of helminth status; 66 were helminth/HIV co-infected and 31 were HIV positive helminth uninfected study participants (Fig. 1). Of helminth/HIV co-infected, 40, 16, and 10 study participants who had intestinal helminths were obtained from Chencha, Arba Minch and Gidole hospitals respectively.

Study participants who did not attend appointments were tried to trace at their homes by the staff of the coordinator of “People Living with HIV/AIDS (PLWA)” and they were encouraged to attend the next follow up session based on the appointment. Eligible study participants were assigned into different groups based on helminth status: helminth/HIV co-infected and helminth uninfected/HIV-positive. The Pre-ART helminth/HIV co-infected study participants CD4+ count were recorded at baseline, 15 weeks and 6 months. They were also treated with anti-helminthic treatment and evaluated for re-infection at each intervals, whereas helminth uninfected/HIV-positive Pre-ART individuals CD4+ T-cell count were also followed up at baseline and 6 months.

### HIV status and CD4+ count determination

HIV status was assessed in all participants using current rapid HIV testing algorithm with KHB, STATPAK (CHEMBIO DIAGNOSTIC SYSTEMS, INC.) and Uni-Gold (Trinity Biotech plc. Bray, Ireland) in PIHCT and VCT centers of the hospital. Patients were considered to be HIV infected if two of HIV rapid tests were positive. CD4+ T cells were enumerated in EDTA blood by use of FACS count (Becton–Dickinson San José A, CA, USA). CD4+ count results were taken every 3 months according to their regular follow up in ART clinic and laboratory. Blood sample was collected routinely with similar rhythmic period to the baseline measurement in ART laboratory.

### Stool sample collection and examination

All individuals who had volunteered and fulfilled the eligible criteria were included in examination for parasite infections. On each follow up time in ART clinic study participants provided a stool sample. Stool samples were examined microscopically by direct (saline and iodine mounts) to identify larvae/rhabditiform larvae of *Strongyloides stercoralis* and ova of helminthes. Fecal samples were preserved in sodium acetate Acetic acid Formalin (SAF) solution on which formol-ether concentration technique was applied to optimize helminth parasite detection.

### Data analysis

All raw data collected from this study was entered into Microsoft data base system and referenced with location. This included data on age, sex, and parasitological result, CD4+ T-cell count at three points of time, antihelminthic treatment and control group. The data were analyzed using SPSS version 16.0. Data were analyzed by the use of paired sample-t test and independent sample-t test statistical analysis and ANOVA for comparing means. Values were considered to be statistically significant when P-values are less than or equal to 0.05 in each of the analysis.

### Ethical consideration

The ethical committee of the Department of Biology, College of Natural Sciences, Addis Ababa University, approved the study. Informed consent was obtained from the study participants and from guardians of children. All helminth and protozoan infected individuals were treated free of charge as per national drug policy by attending health professionals in the ART clinics.

### Antihelminthic and anti-protozoa treatment

Treatment was given as follows: (1) albendazole (single 400-mg dose) for infection with *Ascaris lumbricoides,Trichuris trichiura,* and hookworm; (2) praziquantel (600 mg a single dose) for infection with *Taenia* species (3) albendazole (400 mg twice daily for 3 days) for infection with *Strongyloides stercoralis* (4) metronidazole (750 mg tid for 5–7 days) for pathogenic protozoal infections, including *E.histolytica* and *Giardia lamblia*; and (5) tinidazole 2 g p.o stat for 3 consecutive days for *Giardia lamblia*.

## Results

### Description of the study participants

The median age of helminth/HIV co-infected Pre-ART individuals at recruitment were 31.53 years (range, 12–82 years); 34 (51.5 %) study participants were female and 32 (48.5 %) were male. The median age for HIV-positive helminth uninfected control group at enrollment was 32.58 (range, 15–60 years); 13 (42.0 %) were males and 18 (58.0 %) were females. The median age of study participants in the two groups were similar (31.53 vs. 32.58 years) (Table 1).

**Table 1:** The composition of Pre-ART HIV-positive study participants and controls with regard to gender and age from Arba Minch, Chencha and Gidole hospitals, Ethiopia, Dec. 2008–Sept. 2009

Study groups	Sex		Median age (range)	Total (n = 97)
	Male (n = 45)	Female (n = 52)		
Helminth(+)/HIV (+) study participants	32 (48.5 %)	34 (51.5 %)	31.53 (12–82)	66
HIV(+)/helminth(−) study participants	13 (42.0 %)	18 (58.0 %)	32.58 (15–60)	31

CD4+ T-cell count at recruitment was >200 cells/μl for helminth/HIV co-infected Pre-ART individuals. CD4+ T-cell count of the study participants were between 217 cells/μl and 880 cells/μl and mean CD4+ T-cell count was 422 cells/μl for helminth/HIV co-infected Pre-ART individuals at enrollment. CD4+ T-cell count of HIV-positive helminth uninfected control group was between 224 cells/μl and 878 cells/μl and the mean CD4+ T-cell count was 384.26 cells/μl at enrollment. There were no statistically significance differences in baseline characteristics between the helminth/HIV co-infected and helminth-uninfected HIV positive group (p > 0.05) (Table 2).

**Table 2:** Baseline CD4+ T cell level of HIV-positive Pre-ART study participants in reference to an overall antihelminthic treatment and control group from Arba Minch, Chencha and Gidole hospitals, Ethiopia, Dec. 2008–Sept. 2009

Study groups	Base line Mean CD4+ ± SD			P value
	Mean CD4+ ± SD	Male	Female	
Helminth/HIV infected Pre-ART (n = 66)	422.7 (152.2)	381.31 (114.78)	436.0 (165.0)	**0.2**
Helminth uninfected HIV + Pre-ART (n = 31)	384.26 (140.0)	351.8 (117.1)	418.1 (157.7)	

### Distribution of intestinal helminthes

In this study, one or more intestinal helminth parasite was detected among the total helminth screened study participants. An intestinal helminth infection was more frequent in all study hospitals and highly prevalent in Chencha hospital. Of 66 helminth/HIV co-infected individuals, there were 42 (63.6 %) *Ascaris lumbricoides*, 8 (12.1 %) Hook worm species, 3 (4.5 %) *Strongyloides stercoralis, Trichuris trichiura* and *Taenia* species 1 (1.5 %) each and 11 (16.7 %) was mixed infection of intestinal helminths. The distribution of intestinal helminth parasites were found 40 (60.6 %), 16 (24.2 %), and 10 (15.2 %) in Chencha, Arba Minch and Gidole hospitals respectively. Among intestinal helminth/HIV co-infected Pre-ART study participants, 11 (16.7 %) were infected with more than one intestinal helminths. The other most frequently detected intestinal helminth was hookworm 8 (12.1 %). Mixed infection of ascariasis and trichuriasis were also more frequent among the study participants. Among the intestinal helminth parasites, single parasite infection with *Taenia* species and *Trichuris trichiura* were lower in distribution in all hospitals. Generally, intestinal helminth infections were found to be highly distributed in the study participants of all study sites (Table 3).

**Table 3:** The distribution of intestinal helminths among HIV-positive Pre-ART study participants from Arba Minch, Chencha and Gidole hospitals, Ethiopia.(Dec. 2008–Sept. 2009)

Intestinal helminths	Hospitals			Total (n = 66)
	Arba Minch hospital (n = 16)	Chencha hospital (n = 40)	Gidole hospital (n = 10)	
	Number (%)	Number (%)	Number (%)	Number (%)
*Ascaris lumbricoides*	9 (56.2 %)	24 (60.0 %)	9 (90.0 %)	42 (63.7 %)
Hookworm species	2 (12.5 %)	5 (12.5 %)	1 (10.0 %)	8 (12.2 %)
*Trichuris trichiura*	–	1 (2.5 %)	–	1 (1.5 %)
*Taenia* species	–	1 (2.5 %)	–	1 (1.5 %)
*Strongyloides stercoralis*	2 (12.5 %)	1 (2.5 %)	–	3 (4.5 %)
Mixed infection	3 (18.7 %)	8 (20.0 %)	–	11 (16.7 %)
*Ascaris* + *Strongyloides*	2 (12.5 %)	1 (2.5 %)	–	3 (4.5 %)
*Ascaris* + *Trichuris*	1 (6.2 %)	6 (15.0 %)	–	7 (10.6 %)
*Ascaris* +Hookworm	–	1 (2.5 %)	–	1 (1.5 %)
Total	16 (24.2 %)	40 (60.6 %)	10 (15.2 %)	66 (100.0 %)

### CD4+ T-cell level determinations

Ascariasis, hookworm and mixed infections constituting the most principal intestinal helminth in this study. By considering the highly prevalent intestinal helminths, the baseline mean CD4+ T-cell count in *Ascari*asis, hookworm, mixed and all types’ parasite/HIV co-infection were recorded and compared with different intervals after post antihelminthic treatment.

On the contrary, there were not enough cases detected in other intestinal helminth infections (Trichuriasis, Strongylodiasis and Taeniasis) to evaluate their mean for comparison after 15 weeks and 6 months follow up. Thus, the CD4+ T-cell count change evaluation of these species were included in all types parasite infection category.

### The effect of antihelminthic treatment after 15 weeks

Only 29 helminth/HIV co-infected Pre-ART study participants CD4+ T-cell count were obtained after 15 weeks of antihelminthic therapy to compare at two points of time (at baseline and after 15 weeks). The result revealed that higher CD4+ T-cell count was observed in *Ascaris lumbricoides*/HIV co-infection (P = 0.05) after therapy. In contrast, lowering CD4+ T-cell count was recorded in hookworm/HIV co-infection, but the difference was not significant (P = 0.69). Also, mixed helminth/HIV co-infected study participants CD4+ T-cell count was higher, but the difference was not significant (P = 0.7) after the 15 weeks antihelminthic treatment. However, the comparable groups sample size is very small. Although there was an increase in CD4+ T-cell count (+38.0 cells/µl) in all treated helminth/HIV co-infection, in contrast to the expected benefits of deworming the difference was not statistically significant (P = 0.16) after 15 weeks of antihelminthic treatment (Table 4).

**Table 4:** Change in mean CD4+ T-cell count of HIV-positive Pre-ART study participants in antihelminthic treatment group after 15 weeks period of treatment from Arba Minch, Chencha and Gidole hospitals, Ethiopia, Dec. 2008–Sept. 2009

Intestinal helminths	Before treatment (at baseline)	After treatment	Differences	P value
Antihelminthic treatment	(n = 29)			
*Ascaris lumbricoides*	n = 24	n = 24		
CD4+ count (cells/µl) (SD)	392.7 (140.0)	456.5 (228.6)	+65.3 (149.4)	P = 0.05
Hookworm species	n = 2	n = 2		
CD4+ count (cells/µl) (SD)	655 (325.6)	503 (75.36)	−151.5 (289.9)	P = 0.69
Mixed infection^a^	n = 2	n = 2		
CD4+ count (cells/µl) (SD)	304.0 (14.1)	372.0 (243.2)	+68.0 (257.0)	P = 0.7
All treated infection^b^	n = 29	n = 29		
CD4+ count (cells/µl) (SD)	406.79 (162.1)	453.82 (217.7)	+48.0 (178.7)	P = 0.16

^a^More than one intestinal helminth infections

^b^CD4+ T-cell count values of all parasites species included in all treated infection category

### The effect of antihelminthic treatment 6 months later

Of the 66 Pre-ART helminth/HIV co-infected study participants, CD4+ T-cell count record were taken from a total of 43 study participants including the 29 helminth/HIV co-infected individuals who attended 15 weeks follow up at baseline and 6 months after post-antihelminthic treatment to observe the deworming effect on CD4+ T-cells count. Among the participants, there was 3 (4.5 %) re-infection status observed in the study. The result revealed that antihelminthic treated participants showed an increase of CD4+ T-cell count in *Ascaris lumbricoides*/HIV co-infection with significant difference(P = 0.02). Similarly, all helminth (multiple infection) treated participants also showed (P = 0.005) rising of CD4+ T-cell count after 6 months of post-antihelminthics treatment. In contrast to the expected outcome, hookworm/HIV co-infected antihelminthic treated individuals CD4+ T-cell count were decrease with insignificant difference (P = 0.3). On the other hand, there was declining of CD4+ T-cell count with (P = 0.3) difference comparing between baseline and 6 months later in control group (Table 5).

**Table 5:** Mean CD4+ count of helminth/HIV co-infected Pre-ART study participants at two points (at baseline and 6 months later) of antihelminthic therapy and of control group from Arba Minch, Chencha and Gidole hospitals, Ethiopia, Dec. 2008–Sept. 2009

Study groups	Before treatment (at baseline)	Post-treatment (6 months later)	Difference (cells/µl)	P-value
HIV(+) *Ascaris lumbricoides* (+) (n = 26)	469.1 (157.7)	551.4 (249.9)	+82	0.02
HIV(+) hookworm (+) (n = 7)	483.7 (209.8)	436.0 (215.1)	−47	0.3
HIV(+) mixed infection^a^ (n = 10)	416.4 (130.6)	468.9 (143.6)	+52.5	0.1
All treated participants (n = 43)	464.5 (157.3)	533.0 (215.5)	+68.5	0.005
Control group (n = 31)^b^	384.26 (140.0)	363.52 (178.2)	−20.74	0.3

^a^Mixed infection (included two species Ascariasis + Strongylodiasis/Trichuriasis/Hookworm infection)

^b^Control group consisted of HIV (+) helminth (−) study participants

## Discussions

The high distribution of intestinal helminth infections in the study area was in concurrence with the retrospective data from monthly hospital reports for the region and consistent with WHO (2004) Health Report for Africa and other studies from tropical areas [1]. The prevalence of soil transmitted infections was more prevalent in Chencha and Gidole hospitals which are located among the moist and cold highland areas of Ethiopia. Such environments are known to be favorable to the ova of helminths [2].

*Ascaris lumbricoides*/HIV co-infection was highly prevalent among helminth/HIV co-infected Pre-ART study participants of all study sites. In agreement with other studies from Ethiopian health institutions [10, 13], a high prevalence of *Ascaris lumbricoides*/HIV co-infection was observed among helminth/HIV co-infected study participants. The lower rate of *Ascaris lumbricoides* and other helminth/HIV co-infections in Arba Minch town was to be expected from a dry environment. A low prevalence of ascariasis in the low and dry areas of Ethiopia has been documented by other studies [14]. The present findings were in agreement with Arba Minch hospital report for 2007.

Predominance of mixed infections with ascariasis and trichuriasis was detected in study participants from Arba Minch and Chencha hospitals. The dual existences of the infections were similar to other study [15]. However, predominance of *T. trichiura* infection only was also reported among HIV positive Pre-ART study participants by other studies [9, 10].

The effect of deworming on CD4+ T-cell count in helminth/HIV co-infected Pre-ART individuals was determined after 15 weeks and 6 months post-antihelminthic treatment. In agreement with the findings of the study by Walson et al. [9] in both studies *Ascaris lumbricoides*/HIV co-infected participants showed an increase in CD4+ T-cell count within 15 weeks period and post-antihelminthic treatment. This significant effect of deworming in *Ascaris lumbricoides*/HIV co-infection may explain the reduction of strong Th2 type immunity with antihelminthic therapy; this is because *Ascaris lumbricoides* infection has a remarkable Th2 immunity skewing capacity among soil transmitted helminths [16, 17].

In agreement with this study, a higher CD4+ T-cell count was recorded in all treated study participants for intestinal helminth infections after antihelminthic therapy, likewise the effect of antihelminthic treatment on the improvement of CD4+ T-cell count was shown in all treated participants of other study [15–18]. Although other factors that could potentiate the progression of HIV to AIDS may exist, this could be because of the enhancement of strong Th2 type immune response following antihelminthic therapy for multiple helminth infection.

The lower CD4+ T-cell count value in hookworm/HIV co-infection was also recorded in the study participants after 6 months post-anhelminthic treatment. This result was not consistent with other recent findings of Walson et al. [9] who showed the expected benefits of deworming of hookworm infection, however the difference was not significant(P = 0.3) after antihelminthic therapy. Similarly, there was no statistically significant (P = 0.16) CD4+ T-cell count change between baseline and after 15 weeks visits in all treated helminth/HIV co-infected. Other confounding factors which affect the deworming benefit such as time, different geographical areas, nutrition and unspecific type of antihelminthic treatment regimens may be considered the result to be inconsistent with other studies. But in agreement with other studies [9, 16] significant change in CD4+ T-cell count was observed in all treated helminth/HIV co-infected Pre-ART individuals (P = 0.005) 6 months after post antihelminthic treatment.

Altogether, the data may provide evidence that shows the value of deworming improved the CD4+ T-cell count and thus delays the spread and the progression of individuals into AIDS [19]. The results also suggest that antihelminthic treatment has positive value in preventing species-specific parasite modulation of immune activation in HIV infection with respect to ascariasis/HIV co-infection 6 months after antihelminthic therapy.

Other studies [9, 20] also showed that Th2 subset of CD4+ T-cells dominate a chronic helminths infection and facilitate HIV disease progression with Th1 suppression in helminth infections. In addition, the predominant Th2 cytokines in intestinal helminth environment have been reported to induce apoptosis of HIV infected and uninfected CD4+ T-cells in the gut [9]. Therefore, this mechanism is considered to account for CD4+ T-cell depletion of helminth/HIV co-infected individuals [9, 21].

The evaluation of CD4+ T-cell count of helminth/HIV co-infection treated versus HIV-positive helminths un-infected Pre-ART study participants indicated that antihelminthic treated individuals relatively demonstrated higher level of improvement of CD4+ T-cell count than helminth uninfected Pre-ART individuals after antihelminthic therapy (p < 0.005 vs. p > 0.05). Therefore, this study has provided some evidence that, deworming in helminth/HIV co-infected individuals would delay the time that the HIV positive individuals would be placed under Pre-ART by preventing depletion of CD4+ T-cells [22]. Unavailability of CD4+ count machine in Chencha and Gidole hospitals, loss of follow up, absence of viral load test and parasite egg counts were the limitations of this study.

## Conclusion

The study showed that treatment of ascariasis had a significant effect on CD4+ T-cell count increase in the treated Pre-ART *Ascaris lumbricoides*/HIV co-infected individuals; whereas the same positive effect was not evident for other intestinal helminth/HIV co-infection detected in this study. In conclusion, this finding on *Ascaris lumbricoides*-specific nature of immune interaction in helminth/HIV co-infection may partly explain the inconsistent reports on the role of intestinal helminths on progression of HIV infection to AIDS. Therefore, a well-designed longitudinal study on helminth species-specific HIV/helminth co-infection will be needed to fully establish the possible benefits of deworming in intestinal helminth/HIV co-infection.
